# An Integrated Approach Using HLAMatchmaker and Pirche II for Epitopic Matching in Pediatric Kidney Transplant—A Romanian Single-Center Study

**DOI:** 10.3390/children10111756

**Published:** 2023-10-30

**Authors:** Paul Luchian Aldea, Maria Diana Santionean, Alina Elec, Adriana Munteanu, Oana Antal, Luminita Loga, Tudor Moisoiu, Florin Ioan Elec, Dan Delean, Bogdan Bulata, Andreea Liana Rachisan (Bot)

**Affiliations:** 1 Clinical Institute of Urology and Renal Transplantation, 400006 Cluj-Napoca, Romania; luchian97@yahoo.com (P.L.A.);; 2 Department of Mother and Child, Iuliu Hatieganu University of Medicine and Pharmacy, 400347 Cluj-Napoca, Romania; dsantionean@gmail.com; 3 Department of Urology, Iuliu Hatieganu University of Medicine and Pharmacy, 400347 Cluj-Napoca, Romania; 4 Department of Mother and Child, Discipline of Pediatrics II, Iuliu Hatieganu University of Medicine and Pharmacy, 400347 Cluj-Napoca, Romania

**Keywords:** kidney, transplantation, epitope, pediatric, renal, graft, HLAMatchmaker, PIRCHE II, mismatch

## Abstract

(1) Background: Renal transplantation (KT) is the most efficient treatment for chronic kidney disease among pediatric patients. Antigenic matching and epitopic load should be the main criteria for choosing a renal graft in pediatric transplantation. Our study aims to compare the integration of new histocompatibility predictive algorithms with classical human leukocyte antigen (HLA) matching regarding different types of pediatric renal transplants. (2) Methods: We categorized our cohort of pediatric patients depending on their risk level, type of donor and type of transplantation, delving into discussions surrounding their mismatching values in relation to both the human leukocyte antigen Matchmaker software (versions 4.0. and 3.1.) and the most recent version of the predicted indirectly identifiable HLA epitopes (PIRCHE) II score. (3) Results: We determined that the higher the antigen mismatch, the higher the epitopic load for both algorithms. The HLAMatchmaker algorithm reveals a noticeable difference in eplet load between living and deceased donors, whereas PIRCHE II does not show the same distinction. Dialysis recipients have a higher count of eplet mismatches, which demonstrates a significant difference according to the transplantation type. Our results are similar to those of four similar studies available in the current literature. (4) Conclusions: We suggest that an integrated data approach employing PIRCHE II and HLAMatchmaker algorithms better predicts histocompatibility in KT than classical HLA matching.

## 1. Introduction

It is well known that a kidney transplant is the most effective treatment for individuals grappling with end-stage renal disease, offering advantages regarding life expectancy and living standards compared with methods of extrarenal epuration. Given that pediatric patients typically undergo their initial transplant at a tender age, it is worth noting that a considerable portion of this demographic may find themselves in need of subsequent grafts over the course of their lifetime. This arises due to the unique challenges and growth factors associated with pediatric transplant recipients, necessitating ongoing medical interventions to address evolving health needs and ensure optimal long-term outcomes. Considering the complications associated with the subsequent development of the de novo donor-specific HLA antibodies (dnDSA), a more detailed evaluation of immunological compatibility between donors and recipients must be obtained [1,2]. Numerous factors have been identified as potential contributors to the emergence of de novo HLA antibodies. These factors cover a wide spectrum, including HLA class II incompatibilities, young recipient age, inappropriate immunosuppression regimens and instances of non-adherence to prescribed treatment protocols [3,4]. The current understanding is that human leukocyte antigen antibodies are directed against epitopes [5]. While an epitope refers to the specific segment of an antigen that interacts with a distinct antibody or T-cell receptor, an eplet is designed to represent the smallest functional unit responsible for determining antibody specificity. It constitutes a smaller portion, measuring approximately 3 angstroms in diameter, within the more prominent overall epitope, which has a diameter that is three times bigger. The eplet structure offers a comprehensive and appealing framework for the description of the entire spectrum of HLA epitopes that hold significance in the context of transplantation. Nevertheless, it is crucial to underscore that only a limited subset of these eplets have undergone rigorous validation to establish their capacity to elicit alloantibody responses. The intricacies surrounding the biological and clinical implications associated with each eplet description remain a subject of ongoing investigation, necessitating further exploration to attain a more comprehensive understanding of their true significance in transplantation [6,7]. The indicator of HLA epitopes and the humoral alloimmune response can be determined by the HLAMatchmaker algorithm, based on appropriate plots of antibody-accessible polymorphic amino acid residues (eplets) present on the HLA of the donor [8]. An additional predictive tool for anticipating the alloimmune response subsequent to transplantation is the PIRCHE-II algorithm [9], which predicts donor-HLA-derived peptides presented by the recipient’s HLA-DRB1 molecules. Although each of these algorithms employs its unique approach, they all share a common principle: the likelihood of a more robust recipient T/B-cell response increases as the donor becomes more genetically distinct from the recipient. In consequence, the program produces a quantified outcome that measures the extent of disparities existing between the donor and recipient, thereby simplifying the evaluation of potential immune responses [10,11]. Our study aims to integrate an evaluation of the efficacy and limitations of HLAMatchmaker and PIRCHE as independent predictors of donor–recipient HLA mismatches with an investigation into the epitopic load in relation to antigen mismatches across various donor–recipient subsets. In doing so, we seek to offer a perspective that could refine the current criteria for donor and recipient selection in organ transplantation.

## 2. Patients and Methods

### 2.1. Patient Population

We methodically reviewed electronic patient records obtained from the Clinical Institute of Urology and Renal Transplant Cluj-Napoca for all consecutive pediatric renal transplantations from 1992 to 2020. We included, in the present study, 55 pediatric patients (30 girls, 22 living donors, with an average age at the time of transplantation of 12.07 ± 3.44 years). Only pediatric patients who had undergone complete HLA typing (including A, B and DR) and were consistently followed up for their medical progress were included in this study. All patients belonged to the Caucasian demographic. For donors, we performed serological typing. HLA typing data that were not available were estimated using HLA-ABCDRDQ haplotype frequency data obtained from the National Marrow Donor Program database in 2007 for individuals of European descent, as documented on www.haplostats.org (accessed on 6 September 2023). The cross-match was negative for all patients and they were all ABO compatible.

### 2.2. PIRCHE Analysis

The most recent iteration of the PIRCHE II algorithm from AG-Berlin, Germany, was used to compute the mismatched peptide epitopes derived from HLA, which can be presented by the recipient’s HLA-DRB1 molecules. www.pirche.com (accessed on 6 September 2023) was used to access the algorithm and the data were retrieved in November 2021. One of these promising theoretical approaches is matching based on the Predicted Indirectly Recognizable HLA Epitopes (PIRCHE-II) algorithm and it has shown the potential to improve graft survival. The score is calculated via the PIRCHE-II algorithm using the theoretical number of the donor’s HLA-epitopes, which contain 9 amino acids that can induce an indirect alloreactive response, specifically involving the CD4+ T-cell recognition of HLA class-II presented donor HLA-peptides [12]. Hence, the algorithm’s aim is to identify HLA epitopes from the donor that can be displayed by the recipient’s HLA-DRB1 alleles, but which are not present in their own HLA-A, -B, -C, -DRB1 and -DQB1alleles. This is a resemblance to the indirect pathway of allorecognition, specifically involving CD4 T cells. The count of 15-mer peptides that originate from HLA proteins encoded in exons 2 to 5 of donor HLA genes is quantified by the PIRCHE II score. The peptides in question are notably absent in the recipient’s self-HLA protein repertoire. However, there is a high likelihood that these peptides are present in the HLA-DRB1 proteins. The indirect allorecognition pathway involving CD4 T cells is largely impacted by this nuanced distinction. For solid grafts, the PIRCHE II describes the number of peptides of HLA proteins encoded in 2–5 exons of the donor’s HLA but not in the recipient’s. Similar to the naturally occurring thymic negative selection process, only candidate peptides proven absent in the patient’s self-peptide background are considered allopeptides [2,13]. In a 2021 study, Unterrainer et al. concluded that PIRCHE-II analysis could already be incorporated into kidney allocation algorithms providing an additional layer of compatibility assessment, particularly in cases where significant HLA incompatibilities exist between the donor and the recipient [14].

### 2.3. HLAMatchmaker Analysis

HLAMatchmaker software (HLAMatchmaker DRDQDP Matching version 4.0. for the first class of eplets (A, B) and version 3.1. for the second class of eplets (DR), available on http://www.hlamatchmaker.net (accessed on 30 November 2021) was utilized to identify potential epitope mismatches between donors and recipients. This relies on two fundamental principles: (1) the immune system’s capacity to recognize and make antibodies for non-self-antigens, particularly epitopes on those antigens, while disregarding self-antigens and epitopes; (2) a small number of polymorphic amino acids near the center of the epitope, which largely establish epitope binding affinity. The evidence and the rationale for these postulates have been reported previously [2,6,7,11]. By inspecting amino acid sequences in donor and recipient alleles, HLAMatchmaker is able to pinpoint and quantify the differences between them. Only polymorphic amino acids hold significance and only amino acids at or near the molecule’s surface accessible to antibody binding are considered. HLA matching suggests a preference for kidney grafts that are well matched, with a minimum of 3 mismatches at HLA-A, -B and -DRB1 compared to poorly matched grafts with 4–6 mismatches at these specific loci. Insufficient immunosuppression can cause HLA mismatches to activate alloreactive T-helper cells that support cytotoxic T cells, B cells and antibodies. This, in turn, produces plasma cells that can harm the transplant. Alloantibodies against highly polymorphic HLA antigens, both pre- and post-transplant, are linked to kidney allograft rejection. The advantages of HLA antigen matching, particularly for kidney recipients from elderly donors, have been highlighted by recent data. This alignment significantly diminishes critical adverse outcomes, including graft loss, rejection episodes, mortality and the onset of non-Hodgkin lymphoma [14]. We identified the subset of epitope mismatches in each donor–recipient pair using the HLAMatchmaker Matching software 4.0.

### 2.4. Statistical Analysis

All results are presented as the mean ± standard error of the mean value. The Student’s unpaired *t*-test was utilized to assess statistical significance, with *p*-values below 0.05 considered significant. Additionally, the strength of the association between the two analyzed parameters was evaluated using Pearson’s and Spearman’s correlation coefficients. Statistical analyses were performed using QI Macros 1997–2021 for Windows (statistical process control software package plugin for creating charts and graphs).

## 3. Results

### 3.1. Descriptive Analysis of the Cohort (n = 55)

**Figure 1:**
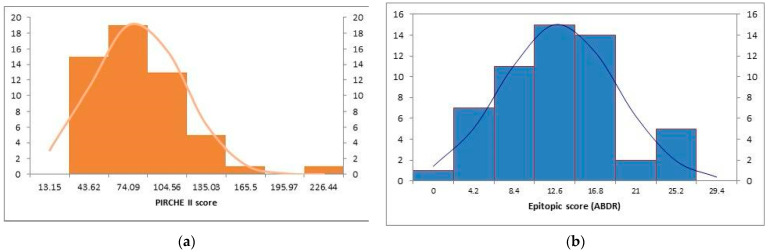
(**a**) Frequency histogram for PIRCHE II score. (**b**) Frequency histogram for epitopic score (HLA matchmaker).

In this cohort of 55 individuals undergoing their first renal transplantations, predominantly from deceased donors (DD) with 33 patients falling into this category, grafts were assigned to recipients with a mean ABDR mismatch of four (ranging from two to six), as detailed in Table 1. The PIRCHE II score had a considerable range of individuals, ranging from 13.96 to 196, with a mean of 66.31 ± 35.66 (as shown in Figure 1a). The HLAMatchmaker determined a range of epitope mismatches, with an average of 10.94 ± 5.98 (as shown in Figure 1b). For the entire cohort, the mean HLA mismatch was four.

**Figure 2:**
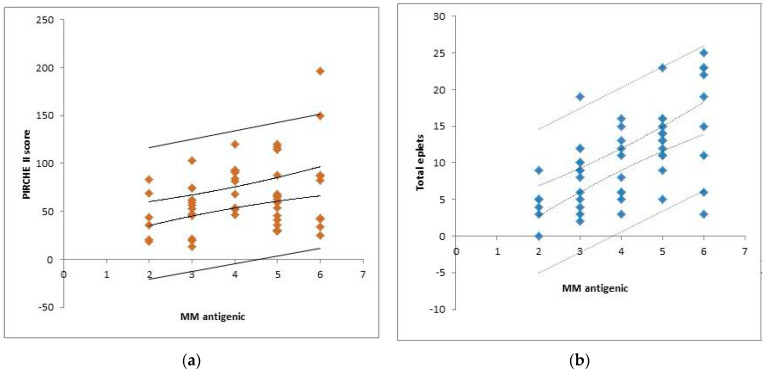
(**a**) Correlation between the antigenic mismatches (MM) and PIRCHE II score. (**b**) Correlation between the antigenic mismatches (MM) and HLA matchmaker score.

The visual representation of Figure 2a shows the correlation between the number of HLA mismatches and the PIRCHE II score (*p* = 0.02, r^2^ = 0.09). Furthermore, the correlation between HLA mismatches and epitopic mismatches, assessed using the HLAMatchmaker, is depicted in Figure 2b (*p* < 0.001, r^2^ = 0.36).

### 3.2. Defining Low-Risk and High-Risk Patients

**Figure 3:**
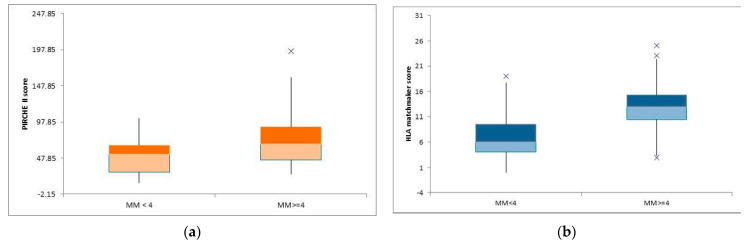
(**a**) Box-plot showing the mean difference of PIRCHE II score between the two subgroups. (**b**) Box-plot showing the mean difference of HLA matchmaker score between the two subgroups.

We created two distinct groups by stratifying the cohort based on HLA mismatches, one that consisted of low-risk patients (HLA mismatches < 4) and the other that included high-risk patients (HLA mismatches ≥ 4). For patients with ABDR mismatches < 4 (n = 19), the mean PIRCHE II score was 50.66 ± 24.77. In contrast, for patients with ABDR mismatches ≥ 4 (n = 36), the mean PIRCHE II score was 74.22 ± 35.37, demonstrating a statistically significant difference with a *p*-value of 0.008 (illustrated in Figure 3a).

In addition, patients with ABDR mismatches < 4 had a mean HLAMatchmaker epitope count of 7.10 ± 4.48, while patients with ABDR mismatches ≥ 4 had a mean HLAMatchmaker epitope count of 12.97 ± 5.98. This difference was also statistically significant, with a *p*-value of 0.001 (depicted in Figure 3b).

### 3.3. Statistical Analysis Depending on Other Parameters (Type of Donor and Transplantation)

**Figure 4:**
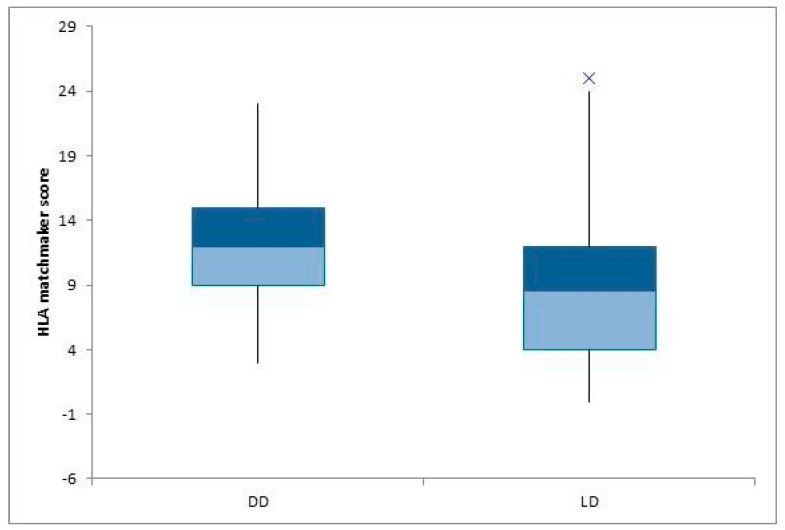
Boxplot showing the HLA matchmaker differences between deceased (DD) and living donors (LD).

**Figure 5:**
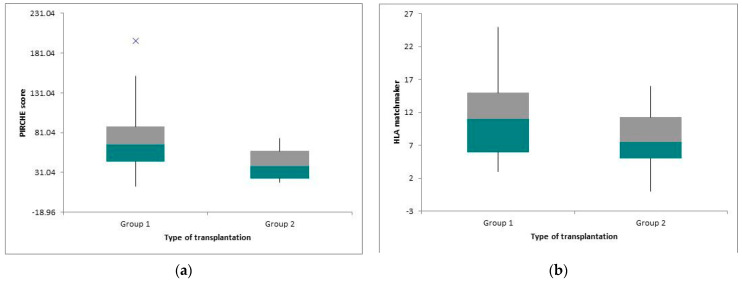
(**a**) Boxplot showing the PIRCHE II differences between the type of transplantation; group 1—non-preemptive, group 2—preemptive. (**b**) Boxplot showing the HLA matchmaker score differences between the type of transplantation; group 1—non-preemptive, group 2—preemptive.

We systematically examined and assessed the cohort regarding the kinds of donors (Table 2). Thirty-three patients received a graft from deceased donors, and four of them (12.12%) had preemptive transplantation. The value of ABDR HLA mismatches was 4.81, and the means of the PIRCHE II score and the eplet incompatibility evaluated using HLAMatchmaker were 71.84 ± 37.56 and 12.33 ± 5.41, respectively. Twenty-two patients received a living donor graft (LD), and six of them (27.27%) had a preemptive transplantation. The number of ABDR HLA mismatches was 3.23, the mean PIRCHE II score was 57.43 ± 30.6, and the mean epitope mismatch HLAMatchmaker score was 8.86 ± 6.33. We noted a statistically significant difference for epitope mismatches calculated with HLAMatchmaker (*p* = 0.04) (Figure 4). A total of 10 patients benefited from preemptive transplantation (18.18%). The value of split HLA incompatibilities was 3.50 ± 1.17, the mean value for the PIRCHE II score was 41.56 ± 19.8, and the mean value for the HLAMatchmaker score was 7.80 ± 5.15. Concerning the non-preemptive group who had extra-renal epuration, either hemodialysis (HD) or peritoneal dialysis (PD), the number of ABDR HLA mismatches was 4.33 ± 1.26, the mean PIRCHE II score value was 71.53 ± 35.88, and the mean HLAMatchmaker score value for eplet incompatibilities was 11.64 ± 5.98. We observed a low epitope load in the preemptive group of patients for PIRCHE II and HLAMatchmaker, with statistically significant differences shown by *p* < 0.01 and *p* = 0.02, respectively (Figure 5a,b).

## 4. Discussion

Our study examined antigenic and eplet incompatibility in a group of pediatric renal graft recipients undergoing their first graft. As per our center’s standard practice, the majority of patients in this study were initially given a standard triple immunosuppressive protocol, which included a calcineurin inhibitor, mycophenolate and steroids. In addition, induction therapy involved administering either polyclonal anti-thymocyte globulin or anti-interleukin-2 receptor antibody. We evaluated the eplet incompatibility using two algorithms: HLAMatchmaker and PIRCHE II. There are only a few studies using the algorithms mentioned above to evaluate the epitopic load in pediatric kidney-grafted patients. In a study published in 2017, Răchisan et al. [15] studied a cohort of 70 children who received grafts from 60 deceased donors; in total, 10% of the patients developed de novo HLA antibodies. The mean follow-up period was 3.5 years, and they used an HLAMatchmaker to evaluate the epitopic load. Branco et al. [16] achieved a five-year survival rate of 84.1% in their study of 124 transplanted children, followed by 10.1 years, and Kausman et al. [17] included in their study 35 patients with a total epitopic incompatibility of 50 eplets and antigenic incompatibility of four whom they followed for one year. Regarding another study, Bryan et al. [18] conducted an evaluation of 16 children who underwent renal graft procedures and determined their eplet load. The median eplet load for DR was 10, and for DQ it was 17. Nevertheless, they did not highlight the presence of dnDSA. The results of the articles are synthesized in Table 3. As demonstrated in previous studies, HLAMatchmaker and PIRCHE are recognized as independent predictors in the formation of donor-specific antibodies (DSA) and graft survival in the context of organ transplantation. This assertion is grounded in scientific research and underscores that these two factors offer distinct and uncorrelated insights when assessing potential compatibility between donors and recipients [19] to explore the epitopic load in relation to antigen mismatches in various subsets of transplants.

While the majority of algorithms designed to match epitopes necessitate high-resolution HLA molecular classification, Geneugelijk et al. [20] has shown that within a Caucasian-only demographic, HLA matching algorithms can still be reliably computed at the split-level HLA antigen designation through statistical interpolation methods guided by haplotype prevalence metrics. We divided the cohort into two groups: low-risk patients (antigen mismatches < 4) and high-risk patients (antigen mismatches ≥ 4), based on the mean HLA mismatches. Several studies also employed a comparable approach to differentiation [21,22]. We propose that, for high-risk patients, the most accurate estimation of epitopic load can be achieved using these two algorithms. Examining this hypothesis, we concluded that the higher the antigen mismatch is, the higher the epitopic load for both algorithms. These findings align with existing data in the published literature.

It is recognized that extracorporeal purification methods prior to transplantation have a negative impact on survival outcomes; this is mainly due to the associated morbidities [23]. Although the immunological aspects involved in dialysis have been studied [24], the current literature lacks comprehensive data supporting a definitive link between preemptive kidney transplantation and the subsequent development of dnDSA. In addition to determining whether predictive scores can provide indications concerning the risk of immune-mediated rejection, we observed that dialyzed recipients exhibited a higher incidence of eplet mismatches, an aspect that was also evident in the analyses conducted through the HLAMatchmaker and PIRCHE methodologies. Confirmation is required using a larger sample and subsequent correlation with the emergence of DSAs.

When it comes to the type of donor, the merits of utilizing living donors for organ transplantation are evident across various dimensions, including logistical optimization and the minimization of cold ischemia intervals, among other factors. However, the choice between a less histocompatible living donor and a HLA-matched cadaveric donor remains a subject of ongoing debate in the medical community. In a comparative study over a 5-year period conducted by Marlais et al. [21] involving pediatric patients, the 5-year renal allograft survival was not inferior for children receiving a kidney transplant from a poorly HLA-matched living donor compared to those receiving a kidney transplant from a well-HLA-matched deceased donor. Opelz et al. [22] showed a better 10-year graft survival in the group of DD with 0–1 HLA mismatches than in the group of LD with 4 to 6 HLA ABDR mismatches. Over the course of the last decade, Kidney Paired Donation (KPD) has emerged as a rapidly expanding and increasingly significant source of kidneys available for transplantation. This innovative approach has effectively addressed a longstanding challenge faced by living donors who were initially considered incompatible with their intended recipients [25]. Despite the successful resolution of barriers encountered by KPD programs, such as ethical and legal complexities, logistical challenges, financial constraints and cultural nuances, in numerous other states, Romania faces a substantial journey before the feasible implementation of such a program. Considering these factors, within our study cohort, living donors consist only of genetically related individuals who demonstrate a significant degree of genetic congruency. The mean frequency of HLA mismatches was observed to be lower in this subset of living donors and our endeavor was to explore whether the difference regarding the HLA mismatches could be extended to the metrics of HLA analysis as well. There was only a significant difference in eplet load between living and deceased donors for the HLAMatchmaker algorithm but not for PIRCHE II. Comparative datasets were not available for analysis. The main reason may be the accuracy of the HLAMatchmaker in comparison with PIRCHE II, which uses only the HLA DRB1 molecules, despite extensive heterogeneity. At the time of the study, the Euro Transplant Kidney Allocation System (ETKAS) considered only the ABDR mismatches [26]. Similar ranges of PIRCHE scores were observed in earlier findings that were limited to DRB1 alone [19,27]. Higher ranges in the PIRCHE score were observed in the study conducted by Shintaro et al. [28], attributable to the inclusion of multiple HLA loci—specifically DRB1 3 4 5 si DQB1—for consideration in the analysis, suggesting that typing only for DRB1 has several limitations in providing a complete picture of the risk of rejection or compatibility. In addition to this, it has been noted in clinical practice that rejection cases conforming to the histological criteria for Antibody-Mediated Rejection (AMR) while lacking detectable anti-HLA DSA make up a significant portion of rejection incidents. These situations may be clarified by the presence of antibodies generated against histocompatibility antigens unrelated to HLA. The Major Histocompatibility Complex Class I-Related Chain A (MICA) antigen, situated near the HLA-B locus, is regarded as the most resilient non-HLA polymorphic antigenic system [29]. MICA can be found in normal kidney tissues, where it is expressed in both endothelial cells and podocytes [30]. Raphael Carapito at al. [31] determined that kidney transplants from donors with MICA mismatches are associated with an elevated risk of graft failure, underscoring the significance of MICA compatibility in donor selection. Multiple studies, including the one led by Yizhou Zou et al. [32], have yielded results aligning with this.

The limitations of our study were our small sample size and restriction to a Caucasian population, potentially limiting the generalizability of our findings. We employed extrapolation methods to obtain eplet mismatch loads. Additionally, our serological typing focused solely on HLA ABDR antigens. Lastly, the absence of DSA screening prevented us from establishing a direct correlation between the formation of dnDSA and non-preemptive transplantation. These limitations demonstrate a key research void that future investigations should address.

## 5. Conclusions

In our study, both HLAMatchmaker and PIRCHE appear to provide independent scores that correlate with the number of HLA mismatches. Their presentation provides plausible considerations for future selection criteria in organ transplantation. The potential significance of these scores suggests ways to explore and refine transplantation protocols further. Nevertheless, further investigations are warranted, involving larger cohorts and the examination of the formation of dnDSA across various donor and recipient subsets. Additionally, the limitations associated with focusing solely on HLA-ABDR typing in the context of multiple HLA loci require exploration. The study also provides initial insights into the potential impact of extracorporeal purification methods on donor–recipient compatibility. There should be a rigorous rationale in selecting the graft, even if this implies a more significant time on the waiting list.

## Figures and Tables

**Table 1:** Patient characteristics.

Sex ratio F/M	30/25
Age at the transplantation (years)	12.07 ± 3.44
Preemptive transplantation	10 (18.1%)
Type of dialysis (DP/HD)	15/30
Living donor	22 (12.1%)
Split HLA mismatches (ABDR)	4 (2–6)
PIRCHE II score	66.31 ± 35.66
Split epitopes mismatches (ABDR) (HLA matchmaker)	10.94 ± 5.98

DP = peritoneal dialysis; HD = hemodialysis; HLA = human leukocyte antigen.

**Table 2:** Patient characteristics depend on the type of donor.

	Deceased Donors (DD) n = 33	Living Donors (LD) n = 22	*p* Value
Sex ratio F/M	18/15	12/10	-
Age at the transplantation (years)	11.93 ± 3.85	12.29 ± 2.93	-
Preemptive transplantation	4 (12.12%)	6 (27.27%)	-
Split HLA mismatches (ABDR)	4.81	3.23	
PIRCHE II score	71.84 ± 37.56	57.43 ± 30.6	NS
Split epitopes mismatches (ABDR) (HLA matchmaker)	12.33 ± 5.41	8.86 ± 6.33	0.04

DD = deceased donors; LD = living donors; HLA = human leukocyte antigen; NS = non-significant.

**Table 3:** Literature review of the main published articles.

References	Age at Transplantation	Number of Patients, Sex Ratio	Mean Period of Follow-Up	% dnDSA+	Antigenic Incompatibility	Epitopic Incompatibility	Type of Donor (DD/LD)
Răchișan et al., 2020 [15]	11.2 ± 3.9	70 (44M/26F)	3.5 years	10%	4.98 ± 1.43	15.5	60/10
Branco et al., 2013 [16]	13	124 (70M/54F)	10.16 years	N/A	N/A	N/A	111/13
Kausman et al., 2016 [17]	13.02 ± 1.25	35 (19M/16F)	1 years	N/A	4	50	27/8
Bryan et al., 2016 [18]	14.1 ± 6.8	16 (10M/6F)	N/A	N/A	N/A	10 DR 17 DQ	15/1

% dnDSA+ = percentage of de novo donor-specific antibody; DD = deceased donor; LD = living donor.

## Data Availability

The data presented in this study are available on request from the corresponding author.
